# Behavioral imitation with artificial neural networks leads to personalized models of brain dynamics during videogame play

**DOI:** 10.1162/IMAG.a.1286

**Published:** 2026-07-02

**Authors:** Anirudha Kemtur, Francois Paugam, Basile Pinsard, Yann Harel, Pravish Sainath, Maximilien Le Clei, Julie Boyle, Karim Jerbi, Lune Bellec

**Affiliations:** Computer Science Department, Université de Montréal, Montréal, Canada; Mila – Quebec AI Institute, Montréal, Canada; Centre de Recherche de l’Institut Universitaire de Gériatrie de Montréal, Montréal, Canada; Psychology Department, Université de Montréal, Montréal, Canada

**Keywords:** brain encoding, fMRI, imitation learning, naturalistic paradigms, personalized models

## Abstract

Videogames provide a promising framework to understand brain activity in a rich, engaging, and active environment, in contrast to mostly passive tasks currently dominating the field, such as image viewing. Analyzing videogames neuroimaging data is, however, challenging, and relies on time-intensive manual annotations of game events, based on somewhat arbitrary rules. Here, we introduce an innovative approach using Artificial Neural networks (ANN) and brain encoding techniques to generate activation maps associated with videogame behavior using functional magnetic resonance imaging (fMRI). As individual behavior is highly variable across subjects in complex environments, we hypothesized that ANNs need to account for subject-specific behavior to capture brain dynamics properly. In this study, we used data collected while subjects played Shinobi III: Return of the Ninja Master (an action-platformer released by Sega in 1993), an action-platformer videogame. Using imitation learning, we trained an ANN to play the game while closely replicating the unique gameplay style of individual participants. We found that hidden layers of our imitation learning model successfully encoded task-relevant neural representations, and predicted individual brain dynamics with higher accuracy than models trained on other subjects’ gameplay. Individual-specific models also outperformed several baselines to predict brain activity, such as pixel inputs, or button presses. The highest correlations between layer activations and brain signals were observed in biologically plausible brain areas, that is, somatosensory, attention, and visual networks. This work thus demonstrates that subject-specific imitation models can be trained from scratch and improve brain encoding in an active naturalistic task. Our framework builds on a commercial videogame of unprecedented complexity in the fMRI brain encoding literature. We used a flexible game emulator that supports a broad range of commercial videogames, opening new naturalistic interactive environments for cognitive neuroscience.

## 1 Introduction

Neuroimaging studies of human cognition increasingly rely on naturalistic paradigms that employ dynamic, rich, and complex stimuli (Sonkusare et al., 2019). In most previous works, the tasks studied and stimuli used have been mostly passive, such as watching videos (Eickhoff et al., 2020; Vanderwal et al., 2019), or free reading (Hsu et al., 2019). However, these tasks are still far from the interactive environments in which the brain normally operates. Videogames have been shown to strongly engage participants’ attention, emotions, motor and decision-making abilities (Anderson et al., 2011; Bavelier et al., 2012; Palaus et al., 2017). As such, videogames offer a robust framework to model the integration of brain dynamics throughout the perception-to-action loop (Bellec & Boyle, 2019). However, analyzing neuroimaging data collected during complex videogame play is challenging, as mapping game events with cognition often requires some a priori choice of annotations which are usually generated manually (Mathiak & Weber, 2006; Weber et al., 2006). In this work we explore a new method to characterize brain activity during videogame that leverages the full extent of the game’s complexity by using artificial neural networks (ANNs) and brain encoding (Naselaris et al., 2011).

Brain encoding has emerged as an alternative method to traditional linear general models based on manual annotations for producing brain maps. Brain encoding is especially powerful for naturalistic stimuli, when the features driving brain activity are unknown a priori. In a brain encoding analysis, a computational model is first trained to do a task of interest using a rich sensory data stream as input (e.g., annotation of objects from pixel-level image data), which is then used to predict the brain responses while performing the same task (Schrimpf et al., 2018). Many brain encoding studies have successfully applied deep neural networks to model the functioning of different brain functional systems, such as vision (Hong et al., 2016; Yamins & DiCarlo, 2016), audition (Kell et al., 2018), and language (Caucheteux & King, 2022), leading to a better understanding of the neural basis of these cognitive functions. However, previous applications of ANNs have focused mainly on passive sensory processing and less on active tasks involving decision-making.

A recent work demonstrated that ANNs can successfully be applied to brain encoding in videogames, specifically for classic Atari games (Cross et al., 2021). However, Cross et al. relied on a generic reinforcement learning model whose behavior was not specifically tuned to the subjects’ gameplay. Although this generic model approach may have been adequate for relatively simple games, it does not allow for exploring inter-individual behavioral variations during videogame play. Of particular interest is the fact that individual human videogame play can be rich and diverse given a sufficiently complex environment (Bavelier & Green, 2019; Duarte et al., 2020). There is also substantial empirical evidence showing that functional brain organization differs qualitatively across individuals, even in the absence of pathology. In particular, studies from the Midnight Scan Club have demonstrated this using deep sampling and resting-state connectivity analyses (Gratton et al., 2018). The existence of substantial behavioral and neural idiosyncrasies supports the use of personalized AI-based brain encoding models, tailored to individual playing styles.

**Fig. 1.**
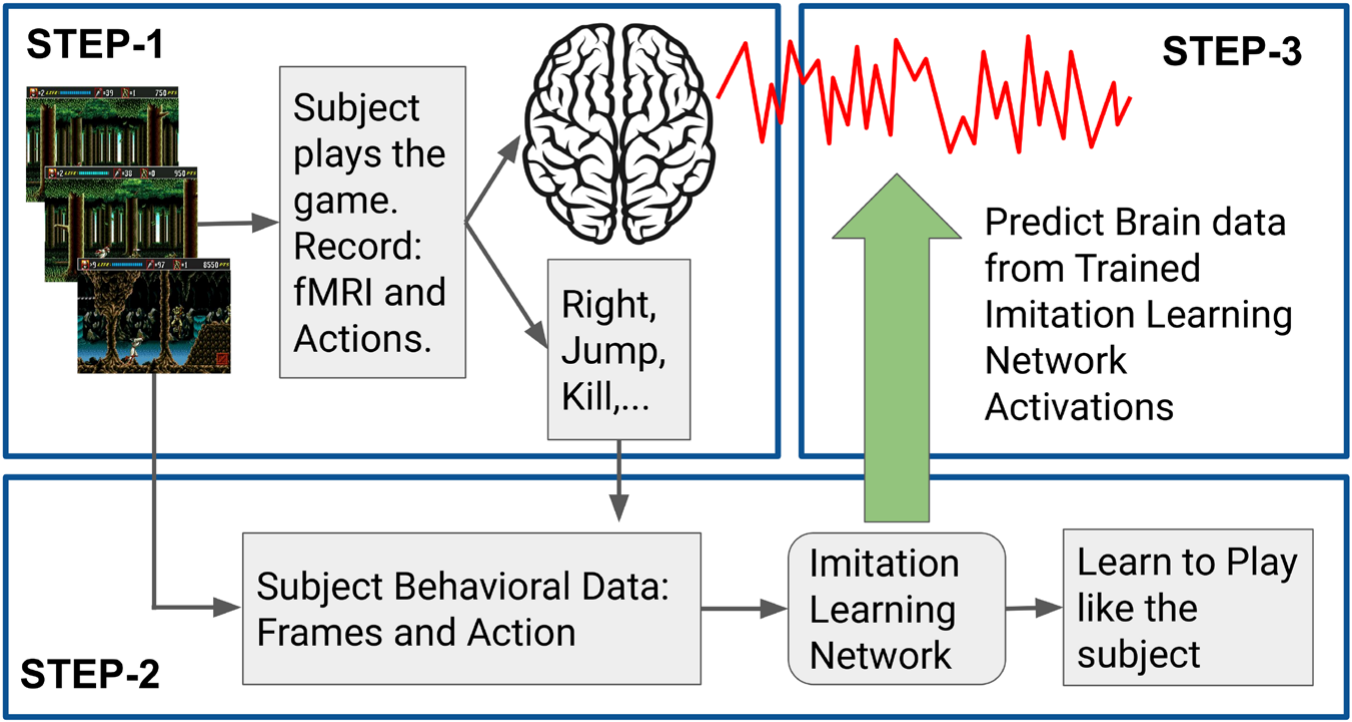
Experiment pipeline. Step-1: Record videogame frames, fMRI data, and key presses of the subject. Step-2: Train an imitation learning neural network using frames and action data, so that it learns to play with a gameplay style similar to the subject. Step-3: Use activations from the neural network model to linearly predict the fMRI data.

In this work, we propose a method to encode brain activity with ANNs during videogame play. Our central hypothesis is that individual behavior is key to training accurate ANN models of brain activity in such environments. Specifically, we test whether ANNs trained to closely imitate individual gameplay in the action game Shinobi III yield more accurate brain encoding models for that individual than models trained on other subjects’ behavior (see Fig. 1 for an outline of the approach). We also compare individual-specific ANNs with several baselines, including button press annotations (capturing fine-grained motor behavior) and vision-based encoding (capturing fine-grained visual input).

Our main contribution is to demonstrate that subject-specific imitation models can be trained from scratch and improve brain encoding in a naturalistic, dynamic task.

Our aim is not to derive new insights into brain function per se, but to establish a methodology and validate an experimental paradigm that can support future research in cognitive neuroscience, particularly studies leveraging rich, interactive environments and capturing idiosyncratic aspects of behavior.

## 2 Materials and Methods

The summary of our experiment pipeline is shown in Figure 1. In the sections below, we describe in detail the dataset and its collection (STEP-1 in Fig. 1), followed by a description of the Imitation learning neural network and its training (STEP-2) and then of how the brain encoding was performed (STEP-3).

### 2.1 Dataset

The authors’ team developed the Shinobi dataset utilized in this study and previously described in detail elsewhere (Boyle et al., 2020; Harel et al., 2026). The dataset consists of both fMRI and behavioral data acquired while participants played the videogame Shinobi III: Return of the Ninja Master (an action-platformer released by Sega in 1993). Data were collected on four right-handed subjects (two women) aged 33 to 49 years during acquisition, labeled as sub-01, sub-02, sub-04, and sub-06. The detailed development and validation of the experimental setup used to acquire this dataset have been extensively documented by the authors in Harel et al. (2023).

#### 2.1.1 Shinobi videogame

Participants played Shinobi III: Return of the Ninja Master, an action-scrolling platformer videogame in which the player controls a ninja character attempting to liberate their village from a fictional group of mercenaries. Three specific levels of the game (levels 1, 4, and 5) were selected due to their similarity in gameplay structure. In these levels, the player begins at the far left of the map and aims to reach and defeat a boss character located at the far right end. Throughout the levels, players can move, crouch, jump, attack enemies, collect bonuses, and avoid obstacles. Actions can be combined to produce special attacks (e.g., low-kick by crouching and attacking at the same time) and interact with the environment (e.g., wall-jump), leading to a variety of possible strategies. Completing a level typically requires approximately 2–3 min, with a level repetition considered complete either upon defeating the boss or upon the player’s character dying. The game was emulated using OpenAI’s gym-retro (version 0.17) (Nichol et al., 2018), a toolbox facilitating the extraction of game frames and memory state variables that supports hundreds of commercial videogames. Visual presentation was controlled using PsychoPy software (Peirce et al., 2019).

Example gameplay runs from all four participants are provided in video link here. These examples illustrate the variety of approaches observed in our study. Subject sub-01 displayed a swift and efficient style, typically defeating enemies with precise jump kicks or sword strikes. Subject sub-02 adopted a similar approach but with less consistent precision in attacks. Subject sub-04 also showed fast-paced gameplay, flexibly employing a mixture of techniques, including jump kicks, sword strikes, and projectile weapons (shurikens). In contrast, subject sub-06 adopted a markedly slower style, often preferring to walk rather than run and relying on ranged attacks such as fireballs or shurikens instead of close-range combat.

#### 2.1.2 Experimental setup

In the first phase of the experiment, participants were instructed to play a customized version of the Shinobi videogame outside of the MRI scanner, using a computer monitor or television. Subjects practiced at home for a minimum of 6 h across all three levels and were considered sufficiently proficient when they were able to complete each of the three levels. However, due to logistical constraints related to the COVID-19 pandemic, the start of the scanning experiments did not immediately follow the time at which subjects reached this level of proficiency. Some participants were also recruited much later than others (sub-04 and sub-06). As a result, the exact amount of practice varied substantially across participants (from 6 h 53 min for sub-06 to 26 h 34 min for sub-02), and their proficiency at the game varied accordingly. For instance, sub-06 began scanning while only able to complete level 4 in less than 15% of attempts, whereas sub-02 completed the same level nearly 30% of the time. The exact training times per subject and per level are available in Table Annex 1 of Harel et al. (2026), and the total amount across levels is included in Table 1 below.

**Table 1.** Phase 1 dataset details.

Subject	Practice time
01	15 h 33 mins
02	26 h 34 mins
04	12 h 42 mins
06	6 h 53 mins

After the training phase, participants were invited to the MRI facility to undertake additional gameplay sessions in phase two of the experiment. During these sessions, behavioral data (controller key presses) and functional MRI (fMRI) blood-oxygen-level-dependent (BOLD) data were simultaneously collected. Approximately 10 sessions per participant were recorded, each session lasting around 40 min and comprising multiple gameplay runs. Each run included sequential repetitions of levels 1, 4, and 5, cycling back to level 1 upon completing all three levels. Each run had a minimum duration of 10 min, concluding at the end of the ongoing repetition after 10 min had elapsed.

Only phase two data (behavioral and fMRI) were analyzed in the present study.

Some runs and sessions were excluded based on quality control assessments for the following reasons: some runs were interrupted by participants, resulting in substantial missing fMRI data; some runs had corrupted log files, preventing access to behavioral data; and recording errors made by the experimenter occasionally led to mismatches between the fMRI and behavioral data.

The data used per subject is shown in Table 2.

**Table 2.** Phase 2 dataset details.

Subject	Total sessions	Total runs	Data (in hours)
01	12	53	7.20
02	8	37	5.41
04	8	40	6.02
06	10	43	6.35

#### 2.1.3 fMRI data

MRI data was collected using a 3T Siemens Prisma Fit MR scanner, a 64-channel head coil. fMRI data were collected using an accelerated simultaneous multi-slice imaging sequence (Setsompop et al., 2012) with a spatial resolution of 2 mm isotropic, and a Repetition Time (TR) of 1.49 s. Visual stimuli were projected onto a screen via a waveguide and presented to participants on a mirror mounted on the head coil. A more detailed description of the MRI and fMRI sequences and setup is available at Courtois Neuromod’s project documentation page: https://docs.cneuromod.ca

#### 2.1.4 Data preprocessing

*Behavioral data* were recorded at 60 Hz. However, it was observed that humans play the game at about 10 to 15 Hz (min press duration of 4–6 samples), which translates to each controller key press being repeated 4 to 6 times in our recordings. Hence, we downsampled the behavioral data to 12 Hz. This resulted in having 18 videogame frames + controller key presses for every 1.49 s (duration of each fMRI TR). Further, we also cropped out the top portion of the videogame frame where the game score/health/ammunitions are displayed.

*fMRI data* were preprocessed using the fMRIprep v20.2.3 LTS (Esteban et al., 2019). A high-pass filter of 0.01 Hertz and spatial smoothing with FWHM = 8 mm were applied to the data, and [motion + wm_csf + global] confounds were then regressed out using nilearn’s load_confounds package. Finally, voxel space fMRI data were converted into parcel space using MIST- 444 atlas (Urchs et al., 2019).

### 2.2 Imitation learning

Imitation learning (IL) is a form of reinforcement learning that resembles human observational learning: having an expert perform the desired behavior and making the model’s policy imitate the expert. In this study, we aim to determine if training a network to mimic the gameplay style of the subject more accurately encodes the subject’s brain data. We use imitation learning to train our behavior encoding model.

#### 2.2.1 Model architecture

**Fig. 2.**
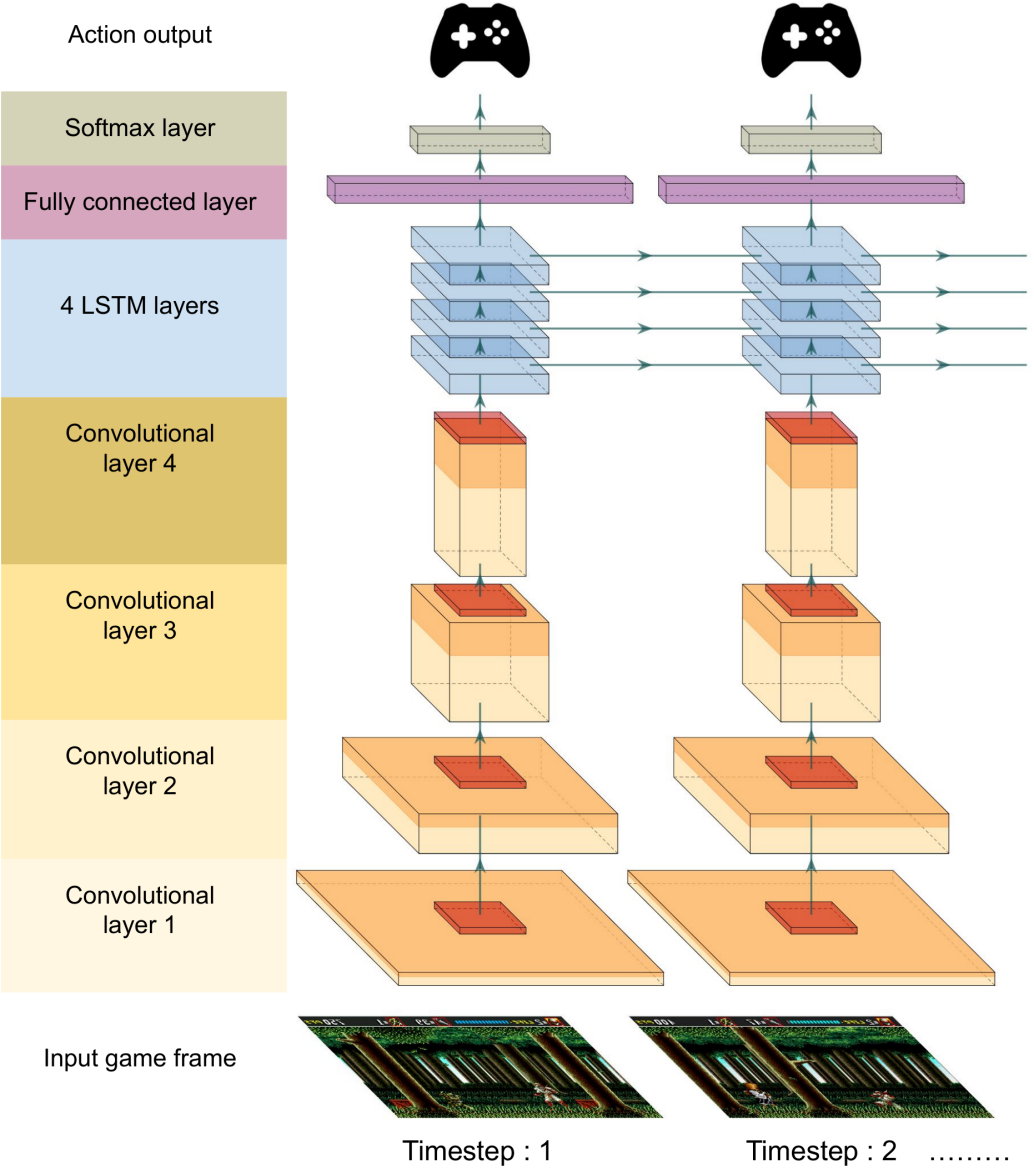
Model architecture is composed of four convolutional layers, four LSTM layers, and a fully connected layer to output softmax probabilities over the action space.

We use a deep neural network-based imitation learning model. The network architecture of our model is shown in Figure 2. As the objective here is to analyze a video game frame and determine the appropriate action to mimic the subject’s behavior, the model must process visual data and maintain a record of actions taken in the preceding timestep. Therefore, our imitation learning model consists of four stacked convolutional layers, followed by four long short-term memory (LSTM) layers. The outputs from the LSTM are then fed into a fully connected (FC) layer, which computes softmax probabilities over the action space. There are 2^7^ = 128 possible actions from all possible combinations of 7 button presses.

The architecture of our imitation model is shown in Figure 2 and layer details are described in Table 3.

**Table 3.** Architecture details.

Layer	Type	Filter size	Number of filters	Output size
0	Input	-	-	[1,50,100]
1	Conv+Relu	[5,5]	32	[32,16,32]
2	Conv+Relu	[5,5]	64	[64,12,14]
3	Conv+Relu	[5,5]	256	[256,4,5]
4	Conv+Relu	[4,5]	200	[200,1,1]
8	Flatten	-	-	[200]
9	4 LSTM Layers	-	-	[200]
10	Fully connected layer	-	-	[64]
11	Softmax layer	-	-	[1]

#### 2.2.2 Training

##### 2.2.2.1 Behavior cloning

The simplest and one of the earliest approaches to IL is behavior cloning (BC). BC aims to learn the expert’s policy by treating it as a supervised learning problem. Given the expert frame-action pairs (image of the game x action taken by the target player), we apply supervised learning by minimizing the loss between the model’s predicted action and the subject’s recorded action for that game frame, treating frame-action pairs as i.i.d. examples. While simple, this method has the advantage of being well-understood and interpretable, allowing us to isolate the contribution of subject-specific visual input and behavior to brain encoding.

##### 2.2.2.2 Loss function

There is a prominent class imbalance in the action sequences, notably, the “go-right” action is the most frequent step (due to the nature of a right-scroller game). A gameplay trajectory requires every action in a particular sequence, and removing some actions to balance classes would result in an entirely different trajectory. We solved this class imbalance with a weighted negative log-likelihood loss function (based on the frequency of each action in the data) so that errors on frequent actions are less penalized than infrequent actions. This allows the model to focus more on the infrequent actions while maintaining the original game trajectory.

##### 2.2.2.3 Implementation

The model was implemented using PyTorch 1.6 with Python 3.6. The loss was then backpropagated through the model, and weights were updated using the Adam optimizer (Kingma & Ba, 2017). Early stopping (Zhang & Yu, 2005) was employed to prevent overfitting and the best model was stored for validation. Hyperparameter selection is important to train a good deep-learning model. We carried out an exhaustive search for the optimal hyperparameters following well-accepted principles and approaches in the field (Smith, 2018). The hyperparameter values found are listed in Table 4.

**Table 4.** Training hyperparameters.

Hyperparameter	Value
LSTM sequence length	18
Batch size	50
Training epochs	3000
Learning rate	0.00005

### 2.3 Brain encoding

To analyze the relationship between the feature space learned by the ANN and the subject’s brain data (Mohsenzadeh et al., 2020; Wang et al., 2015), we performed a brain encoding approach using linear mapping. We deliberately used a simple linear encoding model (ridge regression) to isolate the contribution of learned representations from the behavioral model without introducing additional complexity in the encoding stage.

**Fig. 3.**
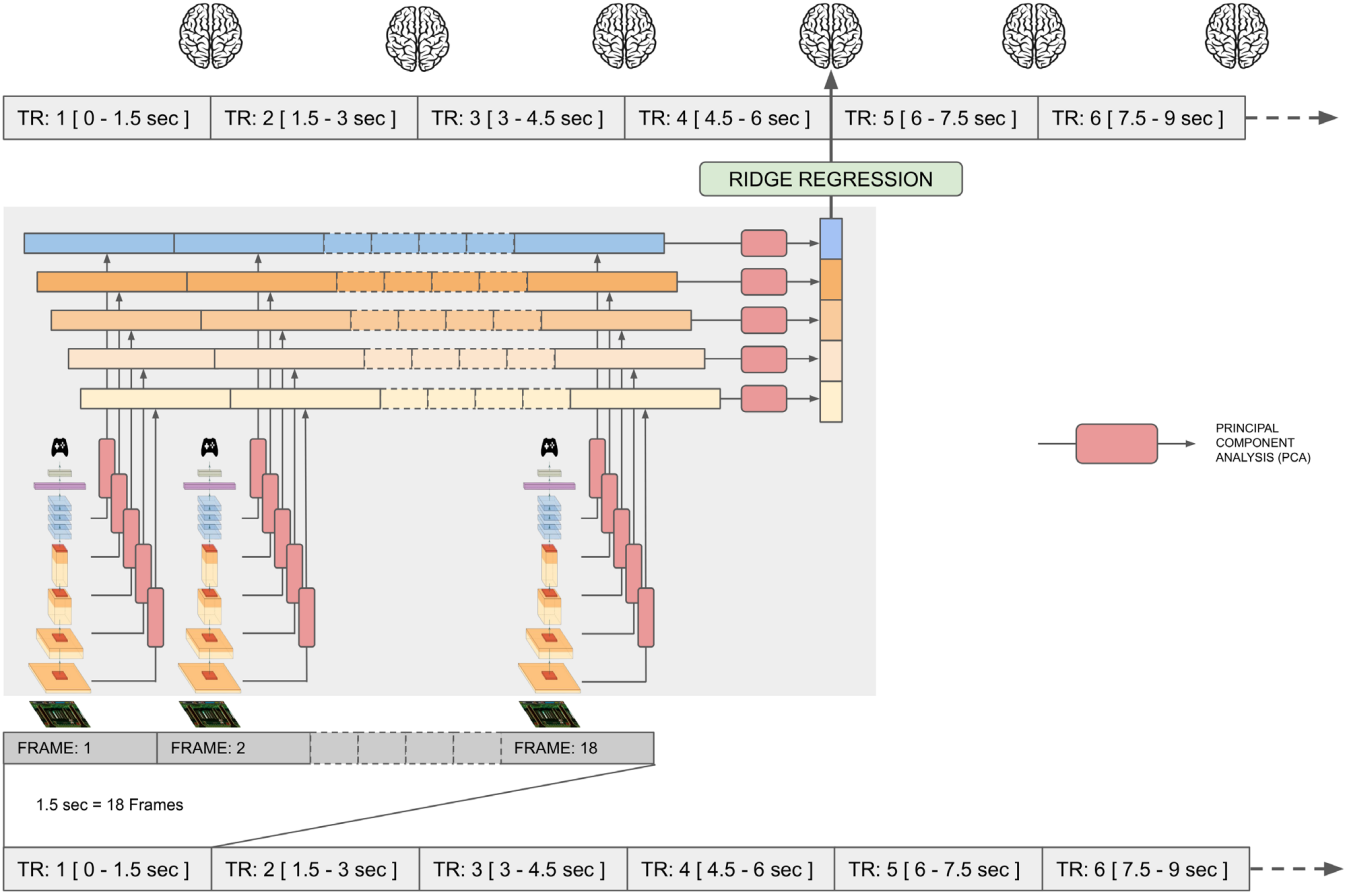
Brain encoding framework. The subject’s gameplay frames are passed into a trained imitation learning model and its internal network activations are reduced in dimension using PCA. These resulting features are used to predict fMRI BOLD responses using ridge regression.

The details of this analysis are summarized in Figure 3. First, we reduced the dimensions of the activations because we have a limited number of data points and cannot use very large feature vector dimensions to train the linear regression model. Activations from the four CNN layers, LSTM layer, and FC layer are reduced in dimension (output vector dimension = 800) using a principal component analysis (PCA). Second, to get encoding features at the fMRI sampling rate, we applied another PCA (output vector dimension = 300) to compress information from 1 TR (1.49 s) windows, independently for each layer. Finally, the PCA vectors resulting from the second PCA from each layer are concatenated to compose the final feature space vector used for ridge regression. To account for the hemodynamic delay, the final feature space vector of the imitation model at time t is mapped to fMRI data at time t+X seconds, where the best encoding was found to be at X=6, consistent with established properties of the hemodynamic response function (Glover, 1999).

Ridge regression was employed to map ANN activations to brain responses. Ridge regression is a regularized variant of linear regression that adds an L2-norm penalty to ordinary least squares (OLS) regression, reducing model complexity and mitigating overfitting. Formally, Ridge regression optimizes the following objective function (Equation 1):

$$\text{Loss}=\;∥\;Y-X\beta \;{∥}_{2}^{2}+\alpha ∥\;\beta \;{∥}_{2}^{2}$$ (1)

where *Y* is the vector of observed fMRI responses for each brain parcel, *X* is the matrix containing ANN-derived feature vectors, β represents regression coefficients, and α is the regularization parameter controlling shrinkage strength.

In this study, Ridge regression models were implemented using the Ridge class from the scikit-learn Python library, employing default solver parameters to ensure stable and efficient computation.

The optimal regularization parameter (α), common for all brain parcels and subjects, was determined through hyperparameter tuning and was found to be 0.5. Separate Ridge regression models were trained for each parcel, allowing brain encoding analysis to capture distributed neural activity patterns across the brain.

### 2.4 Control models

To test whether training an ANN to mimic the behavior of a given individual endows it with higher brain-encoding performance in that same individual, we compared brain encoding performance across a range of control models described below.

#### 2.4.1 Untrained model

When an untrained model processes pixel input, the inherent structure of convolutional and LSTM layers enables the capture of useful features. To assess how much variance is captured solely due to the model’s architecture, we use an untrained model with randomly initialized weights as one of our control models.

#### 2.4.2 Pixel space PCA model

To capture the amount of brain data variance explained just by the videogame frames, we applied a PCA on the pixel RGB input (PCA output vector dimension = 1000) and used it to train the ridge regression model.

#### 2.4.3 Action space model

We use the participant’s key presses to predict the corresponding brain data. This allows us to capture the amount of variance in BOLD signal explained solely by the final action space.

## 3 Results

### 3.1 ANNs trained to imitate individual gameplay successfully encoded brain activity

#### 3.1.1 Behavioral results

We first aimed to train ANNs that could imitate the behavior of individual subjects. We trained a deep ANN composed of vision (CNN) and recurrent (LSTM) layers using behavioral cloning, to predict button presses directly from pixel-level video frames of the game (out of 128 possible actions) (Fig. 2). Leave-one-session-out cross-validation was performed, and the model imitated the subjects’ actions with an accuracy of 62–68%; sub-01: 62%, sub-02: 64%, sub-04: 68%, sub-06: 66% (Chance level: 0.8%). To complement these quantitative performance metrics, we also used trained models to play the game for entire levels, by feeding the actions predicted by the model back into the game emulator. Qualitatively, we found that trained models performed adequately, being able to progress through levels and often complete them, and taking sequences of actions characteristic of individual subjects’ gameplay.

#### 3.1.2 Brain encoding

Next, we tested whether the representations learned by individual imitation models could be used to predict task-related fluctuations in brain activity, for the same subjects. We implemented a brain encoding analysis, where the video frames of a participant’s gameplay were fed into the imitation model. The resulting model layer activations were used as input to a ridge regression that predicted brain activity in all of the brain parcels (Fig. 3).

**Fig. 4.**
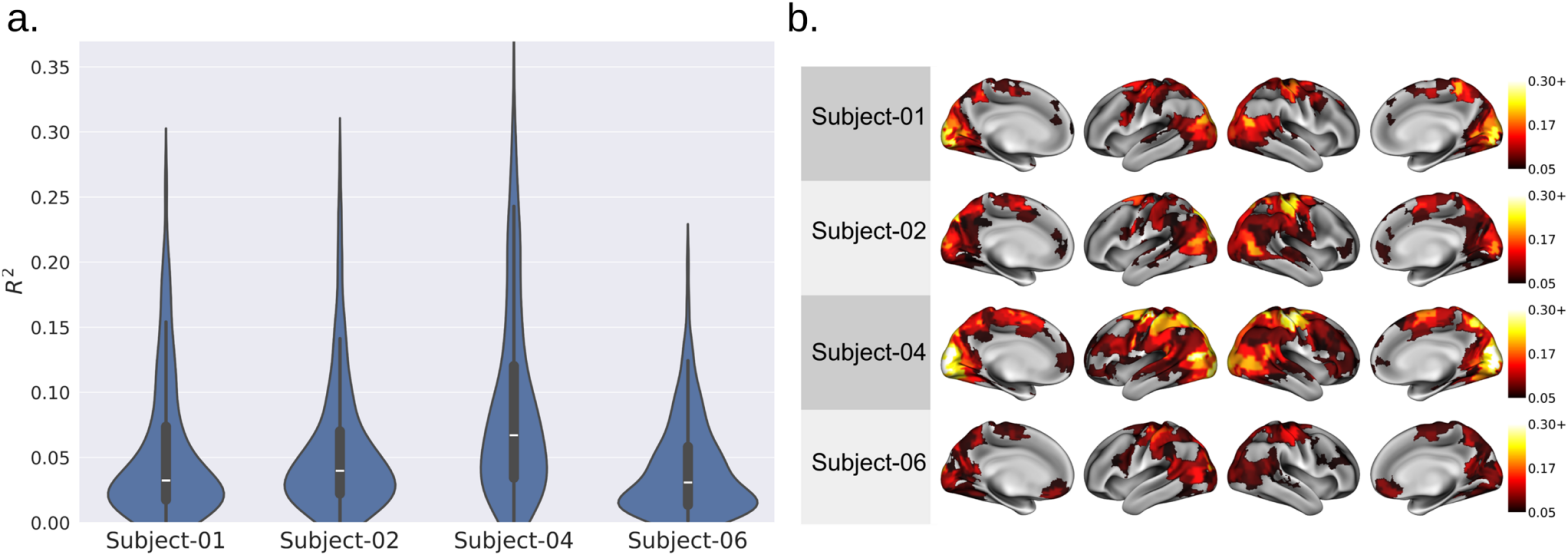
Percentage of variance explained in BOLD data by responses predicted by the model. The fMRI data were predicted from the internal representations of separate imitation learning models, each trained on the corresponding subject’s gameplay. (a) Distribution of brain encoding *R*^2^ (percentage of variance explained in BOLD data by responses predicted by the model) shown across four subjects. For each subject, the distribution of *R*^2^ across 444 parcels is plotted using a violin plot. (b) *R*^2^ across four subjects plotted on a brain map (plotting with an arbitrary threshold of *R*^2^ > 0.05 for visualization purposes)

The coefficient of determination *R*^2^ was calculated between the predicted fMRI data from the ridge regression model and the actual fMRI data for each parcel (Fig. 4). We observe that the imitation learning model predicted brain dynamics with good accuracy in distributed brain regions. Both brain encoding values and the spatial distribution of regions with the highest encoding scores were largely consistent across subjects. Among these, the visual cortex (in particular the dorsal stream), sensorimotor cortex, as well as some frontoparietal areas showed consistently higher prediction, similar to the results obtained by Cross et al. (2021).

#### 3.1.3 Layer wise analysis

In this section, we analyze what effect the early vs later layers of the model have on brain encoding and which brain regions each of them helps to encode better. Apart from the encoding model that uses all the layer features, we consider two additional models. Early layer model, where only the initial two layers (convolutional layer-1, convolutional layer-2) features were used, and the last layer model, where only the last two layers (convolutional layer-4 and LSTM layer) features were used to predict the brain data using ridge regression. This distinction is grounded in findings from the vision neuroAI literature, where early layers in convolutional networks tend to align with early visual areas, while later layers correspond to downstream visual and associative regions (Cross et al., 2021; Yamins & DiCarlo, 2016). In our architecture, the late layers also include temporal recurrence (LSTM), which allows the model to capture behavioral dynamics across time, potentially engaging different brain systems than static frame-based processing.

**Fig. 5.**
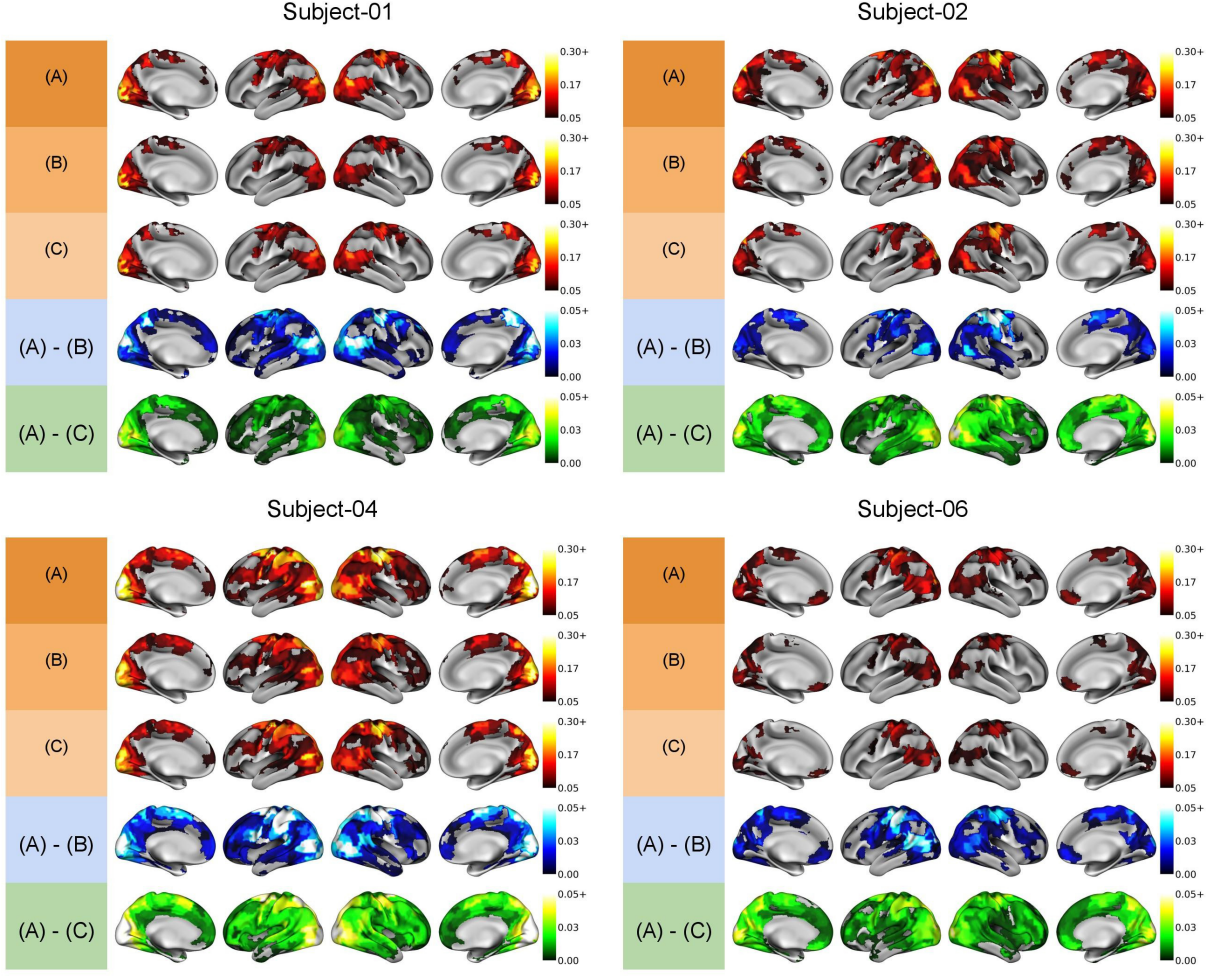
*R*^2^ values on a brain map shown for each of the four subjects. Comparison of encoding based on which layers from the trained model were used. (A) All the layers’ features were used, here displayed with an arbitrary threshold of *R*^2^ > 0.05 for visualization purposes. (B) Only the earliest two convolutional layer features were used **C**: The last convolutional layer and LSTM layer features were used. (A-B) *R*^2^ (All layers) - *R*^2^ (Early layers). This highlights the effect of the later layers (only parcels where the difference is significant at *q* < 0.05 are colored). (A-C) *R*^2^ (All layers) - *R*^2^ (Last layers). This highlights the effect of early layers (only parcels where the difference is significant at *q* < 0.05 are colored).

The *R*^2^ values of these 2 models were projected onto the individual brain, as shown in Figure 5. We found that the early layer better predicted the early visual cortex activation (shown in Fig. 5 by rows (B) and (A-C)), while the last layers of the model better predicted the higher visual cortex, motor, and somatosensory regions (shown in Fig. 5 by rows (C) and (A-B)).

### 3.2 Subject-specific imitation improves brain encoding relative to models trained to imitate other subjects’ gameplay

#### 3.2.1 Behavioral results

**Fig. 6.**
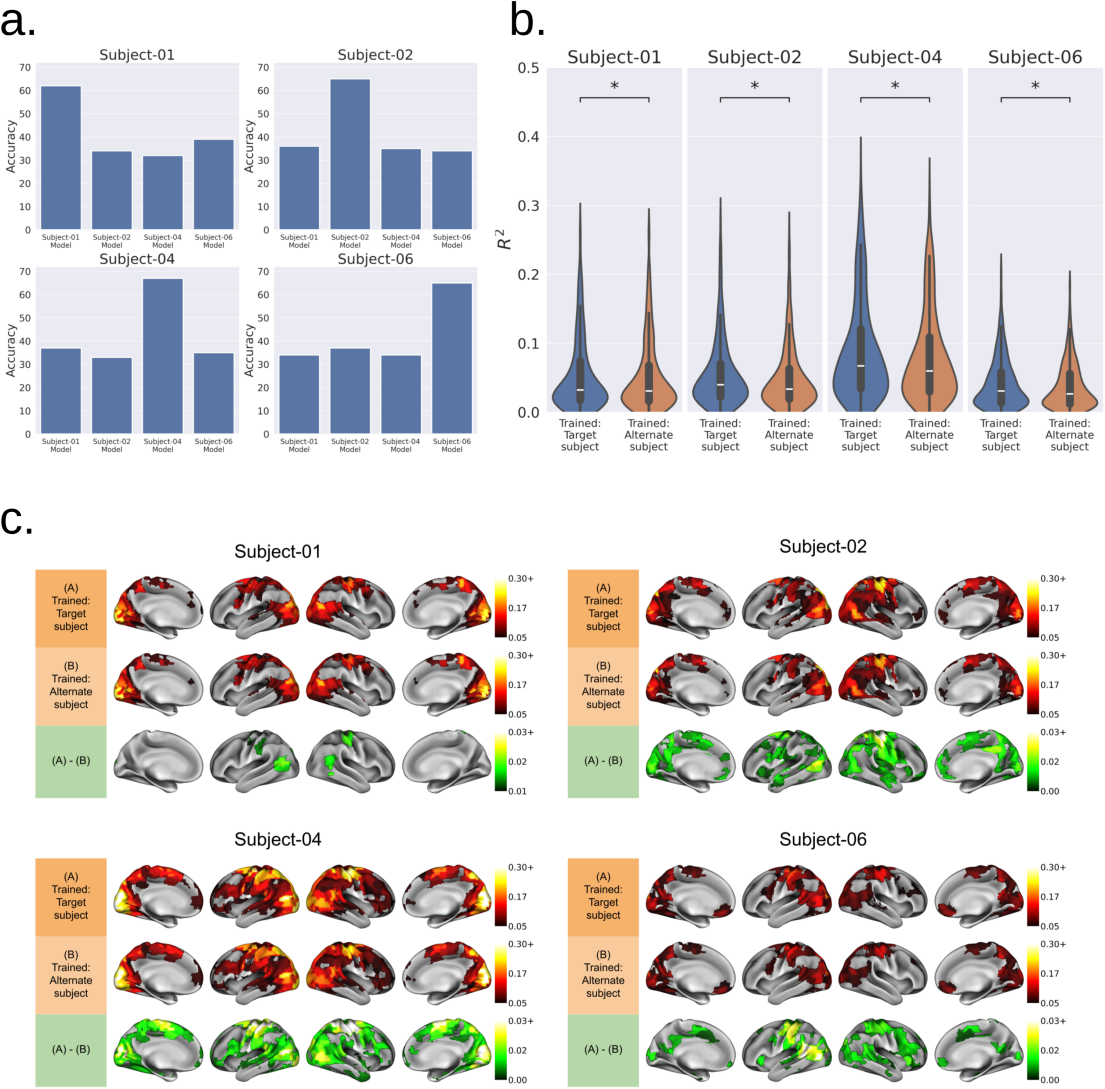
(a) Behavioral imitation learning accuracy across four subjects. X-axis showing which subject the model was trained on. (b) Distribution of brain *R*^2^ comparing encoding performed using a model trained on the target (same) subject vs the alternate (best out of the remaining three) subject. For each subject, significance was tested with a one-sided Mann–Whitney U test on the per-unit difference of *R*^2^; inverted-U brackets indicate the comparison, and asterisks denote p < 0.05. (c) *R*^2^ values on a brain map shown for each of the four subjects. First row: Brain map of *R*^2^ from the trained model (same subject gameplay), with an arbitrary threshold of *R*^2^ > 0.05 for visualization purposes. Second row: Brain map of *R*^2^ from the Trained model (Different gameplay), with an arbitrary threshold of *R*^2^ > 0.05 for visualization purposes. Third row: Brain map of *R*^2^ (trained model: same subject gameplay) - *R*^2^ (trained model: different gameplay), where the *R*^2^ values are subtracted parcel-wise and plotted (only parcels where the difference is significant at *q* < 0.05 are colored).

We saw that the imitation learning model was able to predict behavioral actions with an accuracy of up to 65%. Here, we tested whether these imitation models were subject-specific. Specifically, we evaluated whether the imitation learning model trained on a subject’s gameplay data can model the behavior of that same subject better than a model that knows how to play the game but with a different play style, drawn from the other three subjects. The results showing the within and between subject comparison are presented in Figure 6a. While the imitation learning model trained on the subject’s own gameplay was able to replicate the gameplay data up to 65% accuracy, the models trained on a different gameplay style only achieved 35% accuracy. This result was observed consistently across all four subjects.

#### 3.2.2 Brain encoding

Next, we wanted to see if the subject-specificity of imitation models translated into better brain encoding (higher *R*^2^). Specifically, we tested whether a subject-specific imitation model could encode the brain data of that target individual better than imitation-learning models trained on the gameplay of a alternate subject. This alternative subject was selected as the one most behaviorally similar to the target, that is, whose behavioral model reached the highest accuracy on the target subject (sub-01 was paired with sub-06, sub-02 with sub-01, sub-04 with sub-01, and sub-06 with sub-02). We passed prerecorded gameplay frames of the target subject into these two models: (1) the imitation model trained on the target subject and (2) the imitation model trained on the best behavioral alternative subject. The internal activations from these models were used to predict BOLD activations of the target subject using ridge regression.

Distributions of *R*2 across the parcels for each subject-specific model and the best behavioral alternative model were plotted (Fig. 6b). Across all four subjects, we observed that the target (same-subject) imitation model was better at encoding the brain data than the alternative model selected for that subject.

Figure 6c shows in which brain regions the subject-specific model encoded the brain dynamics better than the best behavioral alternative subject model. These differences were significant at q<0.05 (Wilcoxon signed-rank test with false discovery rate correction for multiple comparisons across parcels). Same-subject imitation learning models better encode brain data than their corresponding alternative subject model in 6%, 35%, 37%, 26% of parcels in Subjects 01, 02, 04, and 06, respectively. Specifically, we found improved encoding in motor, somatosensory, and visual cortices, compared to models trained with different gameplay. These areas are key to processing the input of the task (visual stimuli) and generating outputs (motor commands).

### 3.3 Subject-specific imitation models outperformed a series of control models for brain encoding

We compared brain encoding with subject-specific imitation models to a series of control models: (1) PCA features derived from gameplay input pixel (Pixel-space model); (2) one-hot encoding of the controller button presses (Action-space model); (3) passing the subjects’ gameplay frames to a CNN-LSTM model with random weights (Untrained model).

**Fig. 7.**
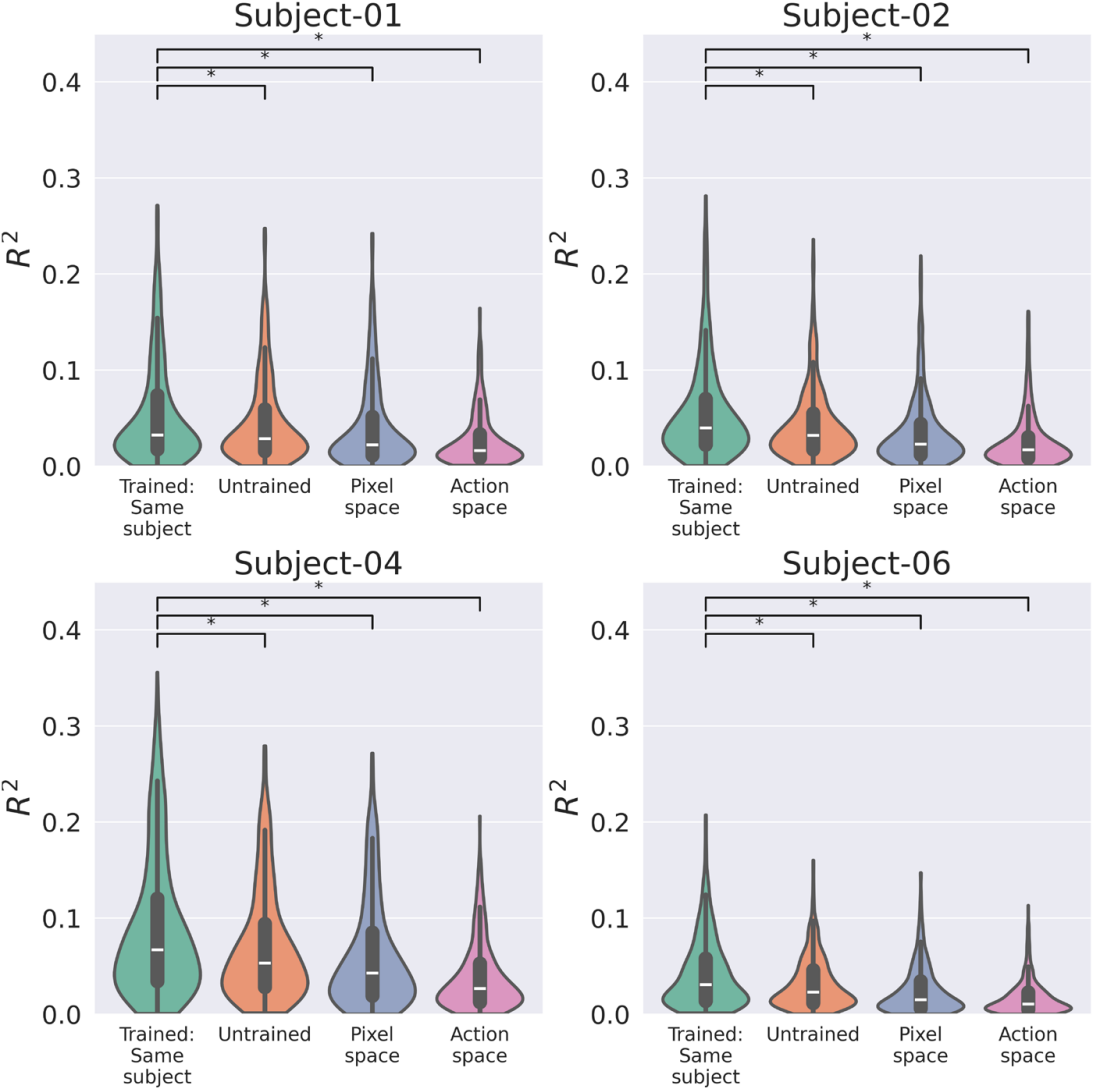
Distribution of brain *R*^2^ comparing encoding performed using a model trained on the target (same) subject vs three control models. Within each subject, we tested whether Trained: Same subject outperforms each other condition using one-sided Mann–Whitney U tests on per-unit *R*^2^ values (H1: Same > Other). Inverted-U brackets indicate the three tested pairs; an asterisk denotes p < 0.05.

Distribution of *R*^2^ across the parcels for imitation and control models was plotted (Fig. 7). Across all four subjects, the subject-specific imitation model was better at encoding brain data than all three controls. Significant improvement in brain encoding using imitation models was found in about 40% of parcels (average across subjects) when compared to the Untrained model, and in about 85% of parcels (average across subjects) when compared with the Pixel-space and Action-space control models.

## 4 Discussion

Our individual imitation models trained with behavioral cloning show good quantitative performance (up to 70% accuracy on a hold-out test set) as well as qualitative face validity when used to play the game autonomously. However, it is difficult to conclude whether these models fully imitate individual gameplay. Humans have variable behavior, and it is very likely that a player would not be able to predict their own actions 100% of the time if presented with recorded videos of their gameplay. Another critical aspect driving variations in behavior is ongoing learning, as subjects get more proficient at the game over time. Unlike simple games like pong, with a clear objective and limited variations in strategies, the Shinobi environment is rich and participants may change strategies over time, for example, favoring speed over health loss, or abandon collecting certain power-ups only to revert to collecting them at a later stage. In this experiment, subjects were extensively trained outside the scanner before neuroimaging sessions to avoid large learning effects. However, given the complexity of the game, learning effects were apparent and variations in strategies still occurred during the imaging sessions (Harel et al., 2026). There were also substantial variations in the amount of practice given to each participant prior to imaging sessions. Our current BC approach did not account for variations in training across participants, or attempt at capturing learning effects during the imaging sessions. We note that more sophisticated imitation learning approaches—such as modeling temporal structure explicitly or learning latent action representations—could better capture trajectory-level behavior, and may be worth exploring in future work. The study by Chen et al. (2021) showed that when trained on various Atari games, the BC approach achieves accuracies between 60% and 90%, depending on the game and the size of training data. The work by Kanervisto et al. (2020) showed that the accuracies of the BC models greatly depend on the quality of data. Hence, it might not be easy to compare our encoding scores to previous works that might have a difference in dataset size, quality, and nature of videogames.

We found that an ANN model trained to imitate the subject effectively encodes their own brain dynamics, with *R*^2^ values up to 0.35. The encoding worked particularly well in motor, somatosensory, and visual cortices. Although these brain regions were consistent across subjects, the average and maximum *R*^2^ varied markedly across subjects. These results likely reflect systematic differences in data quality (and noise ceiling) or individual behavioral consistency across subjects. Noise in fMRI is complex, but a major source is motion (Power et al., 2015), and subjects had markedly different amounts of motion, consistently across scans (Harel et al., 2026). We also found that encoding *R*^2^ was poor in subcortical regions across all subjects, and the sequence used in our experiment had low SNR in those regions, as is common with multiband accelerated fMRI. In raw image presentation, a noise ceiling can be estimated through repeated stimuli presentation to define an optimal brain response, as done in a study by Allen et al. (2022), which was not possible to implement with videogame whose gameplay varied across repetitions and subjects. The only previous study of brain encoding with ANNs in videogames, Cross et al. (2021), achieved a maximum encoding *R*^2^ of 0.38 (average across subjects) and demonstrated the best encoding in visual, motor, and somatosensory areas, results which are similar to ours. It is not possible to directly compare *R*^2^ between the two studies; however, as noise ceiling (and *R*^2^) are directly impacted by parameters such as image resolution, scanning sequence, and the amount of spatial smoothing, none of which were harmonized across the two studies.

To further investigate the functional role of different parts of the models, we compared brain encoding performance using features derived from either the early or late layers. We found that early layers predicted brain activity more accurately in early visual cortex. In contrast, the later layers—which include both higher convolutional layers and the LSTM—showed stronger prediction in higher visual, motor, and somatosensory regions. This is consistent with the idea that early convolutional layers extract low-level visual features, while later layers integrate behavioral and temporal information relevant to action and sensorimotor processing. These results align with prior findings from brain encoding studies using deep networks, such as Yamins and DiCarlo (2016) and Cross et al. (2021).

Further, we found that imitation models were subject-specific. While imitation models trained on individual gameplay were able to predict actions with an accuracy of up to 70% for the target individual, imitation models trained on the gameplay of another subject were only able to predict actions accurately with up to 30–40% accuracy. This improvement in behavioral accuracy also translated to better brain encoding. Specifically, we found that individual-specific imitation models improved encoding in motor, somatosensory, and visual cortices, compared to models trained with different gameplay. These areas are key to processing the input of the task (visual stimuli) and generating outputs (motor commands). While imitation models trained on different play styles can navigate the game, their actions might differ from the target subject, as shown by the loss in accuracy in predicting actions. Representations of these subject-specific actions likely explain the ability of subject-specific imitation models to capture more brain variance in motor and somatosensory areas. By contrast, brain encoding in frontoparietal cortices did not show widespread improvement. Frontoparietal cortices are involved in planning and were expected to show improvement with subject-specific behavioral imitation models. We still note some improvements in the dorsal attentional network, which is a subset of frontoparietal regions tightly linked with motor control and attention. In the experiment setup, we asked the subjects to practice the game for about 6 months before they played the game inside fMRI scanner. They had reached highly consistent gameplay, which might have led to high levels of automation and little effort for planning. This may explain the limited involvement of frontoparietal networks. Using a non-linear brain encoding model is another possible avenue to improve the quality of the brain encoding model, which we actively explore in ongoing work, for example, Freteault et al. (2025), and has also been pursued by other groups in recent years.

Control analysis showed that neither the pixel input nor the player’s actions were sufficient to capture brain dynamics in a complex task like playing Shinobi. We also observe that training an ANN with an imitation model benefits brain encoding, and features derived from an untrained CNN-LSTM network (to which raw input was fed) did not explain any more variance than models trained on raw input game pixel data. These results were observed across all cortical regions. Some previous studies showed that the structure of a CNN network is sufficient to capture a considerable amount of low-level image statistics before any learning (Ulyanov et al., 2018), and such untrained networks can be used to encode the brain in visual tasks (Kim et al., 2021).

Our results show that in complex naturalistic tasks such as videogames, the stimuli or final behavior or network architecture alone are not able to capture the brain dynamics. By contrast, a deep learning model that learns to perform the task similarly to the subject would be able to encode the brain dynamics of that subject more accurately. Cross et al. (2021) study on videogame brain encoding found similar results where raw statistical properties of input/output were not able to explain the variance in brain data well while playing videogames, even if their reference models were not tuned to specific individual gameplay.

One limitation of this study is that, given the slow temporal resolution of fMRI and the rapid dynamics of video gameplay, the advantages of a subject-specific imitation learning model might not have been fully translated into better brain encoding due to a loss of fast action dynamics. Future work using high temporal resolution imaging techniques, such as EEG or MEG, would thus complement this fMRI study.

Our proposed approach to generating brain maps in complex videogames at the individual level opens new avenues for future research. There has been a push toward using more ecologically valid tasks in the context of fMRI research, including to study brain/behavior associations, for example, the Healthy Brain Network project (Alexander et al., 2017). These naturalistic tasks could potentially expose some associations that would otherwise not be potentiated due to a lack of engagement from participants. The most common naturalistic task in fMRI is movie watching, which, while more engaging than resting-state, remains a relatively passive task. The prospect of using commercial videogames combined with ANNs to generate individual brain maps will enable an entirely new class of paradigms to be tested for brain-behavior associations since these maps can directly be entered in a group-level general linear model. Active naturalistic tasks may be particularly well suited for certain demographics, such as children, provided the videogame is selected appropriately for the target group.

States of intense task engagement and the expression of fine psychomotor skill at high levels may be more likely to emerge in naturalistic settings. In this context, video games are known to be potent inducers of psychological flow states (Khoshnoud et al., 2020, 2022), which are notoriously difficult to observe in laboratory tasks (Abuhamdeh, 2020). Our modeling framework does not currently capture within-individual variations in performance, and is therefore not directly suited to studying flow states or other performance changes, for example those related to learning. More complex imitation models could potentially incorporate behavioral non-stationarities, but would likely require individual state annotations or proxies derived from phenomenological reports, physiological measures, or game analytics/ratings, e.g. Sharma et al. (2024). It is also unclear to what extent playing video games in an MRI scanner alters participants’ ability to reach highly engaged states. We thus view our work as an early step towards developing AI models of brain and behavior in naturalistic video game environments.

## 5 Conclusion

In this work, we showed that a deep recurrent neural network is able to successfully imitate the actions of individual human players in a complex videogame and that the representations learned by this network can be used to perform brain encoding. Those derived imitation models were subject-specific, both in terms of behavior (button presses) and brain encoding. Subject-specific imitation models also performed significantly better than a series of controls, including the direct use of button presses to predict brain activity. We found that the improvement in encoding over the control model was particularly noticeable in the task-relevant networks (motor, somatosensory, and visual cortices).

Our methodological framework relies on a flexible game emulator that supports a broad range of commercial games and could therefore be applied to diverse environments in cognitive neuroscience. Such ecologically valid environments have the potential to evoke complex behaviors that are difficult to isolate in traditional controlled laboratory tasks. Our method is thus a step towards modeling complex cognition in ecological environments.

## Data Availability

Data used in the paper can be requested through Cneuromod website: https://www.cneuromod.ca/. The code for the study can be found in a dedicated GitHub repository: https://github.com/anirudhk686/shinobi_imitation_learning.
